# A Tablet App for Handwriting Skill Screening at the Preliteracy Stage: Instrument Validation Study

**DOI:** 10.2196/20126

**Published:** 2020-10-22

**Authors:** Linda Greta Dui, Francesca Lunardini, Cristiano Termine, Matteo Matteucci, Natale Adolfo Stucchi, Nunzio Alberto Borghese, Simona Ferrante

**Affiliations:** 1 Department of Electronics, Information and Bioengineering Politecnico di Milano Milan Italy; 2 Department of Medicine and Surgery University of Insubria Varese Italy; 3 Department of Psychology Università degli Studi di Milano-Bicocca Milan Italy; 4 Department of Computer Science University of Milano Milan Italy

**Keywords:** serious game, tablet, isochrony, homothety, speed-accuracy tradeoff, steering law, writing, prevention

## Abstract

**Background:**

Difficulties in handwriting, such as dysgraphia, impact several aspects of a child’s everyday life. Current methodologies for the detection of such difficulties in children have the following three main weaknesses: (1) they are prone to subjective evaluation; (2) they can be administered only when handwriting is mastered, thus delaying the diagnosis and the possible adoption of countermeasures; and (3) they are not always easily accessible to the entire community.

**Objective:**

This work aims at developing a solution able to: (1) quantitatively measure handwriting features whose alteration is typically seen in children with dysgraphia; (2) enable their study in a preliteracy population; and (3) leverage a standard consumer technology to increase the accessibility of both early screening and longitudinal monitoring of handwriting difficulties.

**Methods:**

We designed and developed a novel tablet-based app *Play Draw Write* to assess potential markers of dysgraphia through the quantification of the following three key handwriting laws: isochrony, homothety, and speed-accuracy tradeoff. To extend such an approach to a preliteracy age, the app includes the study of the laws in terms of both word writing and symbol drawing. The app was tested among healthy children with mastered handwriting (third graders) and those at a preliterate age (kindergartners).

**Results:**

App testing in 15 primary school children confirmed that the three laws hold on the tablet surface when both writing words and drawing symbols. We found significant speed modulation according to size (*P*<.001), no relevant changes to fraction time for 67 out of 70 comparisons, and significant regression between movement time and index of difficulty for 44 out of 45 comparisons (*P*<.05, R^2^>0.28, 12 degrees of freedom). Importantly, the three laws were verified on symbols among 19 kindergartners. Results from the speed-accuracy exercise showed a significant evolution with age of the global movement time (circle: *P*=.003, square: *P*<.001, word: *P*=.001), the goodness of fit of the regression between movement time and accuracy constraints (square: *P*<.001, circle: *P*=.02), and the index of performance (square: *P*<.001). Our findings show that homothety, isochrony, and speed-accuracy tradeoff principles are present in children even before handwriting acquisition; however, some handwriting-related skills are partially refined with age.

**Conclusions:**

The designed app represents a promising solution for the screening of handwriting difficulties, since it allows (1) anticipation of the detection of alteration of handwriting principles at a preliteracy age and (2) provision of broader access to the monitoring of handwriting principles. Such a solution potentially enables the selective strengthening of lacking abilities before they exacerbate and affect the child’s whole life.

## Introduction

### Background

Dysgraphia is a learning disability that involves unsatisfactory handwriting production, given age, normal intelligence, and absence of neurological, perceptual, or motor problems [1]. Dysgraphia can also be characterized by an excessively long execution time notwithstanding adequate readability, resulting in difficulty to keep up with peers in everyday tasks or homework [2,3]. Dysgraphia heavily impacts a child’s school and everyday life, as handwriting is fundamental in the learning process. Handwriting difficulties, especially at an early educational age, have an effect on children’s self-esteem and cause behavioral problems and early school abandonment [4]. Dysgraphia can occur in isolation or in association with a specific learning disorder (eg, developmental dyslexia with a moderate comorbidity correlation) [1] or fall within a more generalized developmental coordination disorder [5]. In the latter case, motor coordination difficulties are not limited to writing, but emerge in other daily activities (eg, playing an instrument, performing sports activities, and lacing up shoes).

The exact epidemiology of dysgraphia is still under debate, resulting in differences in the diagnostic criteria adopted by different schools of thought [6]. Beside the lack of consensus, current diagnostic tools are affected by the following two main limitations: (1) as they are primarily based on handwriting production, the actual diagnosis cannot be performed before complete handwriting maturation in the third year of primary school [7] and (2) the examination itself is based on either qualitative observation of the writing outcome, which might be prone to different interpretations according to the examiner, or the writing speed, which is not a comprehensive indicator.

The limitations of the diagnostic procedure contribute to uncertainty in the estimation of the actual incidence of the phenomenon. Indeed, the reported prevalence of handwriting problems ranges between 5% and 27% [6,8]. Such a wide range includes both underestimation, when a child has no access to a diagnostic visit with a specialist, and overestimation, when the lack of a prompt intervention exacerbates transient difficulties, which are improperly translated into the corresponding disability.

### Needs

Against this background, there is an urgent need for innovative solutions to support the screening for dysgraphia. An effective solution must meet the following requirements: (1) quantitatively reveal potential alterations in writing production; (2) detect potential weaknesses at an early stage, thus enabling early intervention; and (3) be easily accessible to the user in order to reduce delayed diagnoses and allow longitudinal monitoring.

To meet the first requirement, understanding in which way dysgraphia alters the principles of handwriting is fundamental to quantify such alterations. It is known that handwriting problems are often linked to a lacking sense of rhythm [9,10]. In handwriting, rhythm can be translated into the ability of pacing letters to keep constancy in movement execution. Specifically, the following two laws of motion relevant to this scope have been studied during handwriting: *isochrony* and *homothety*. The *isochrony* principle states that bigger gesture execution is accompanied by an increase in average movement speed to keep the movement time approximately constant [11]. The *homothety* principle predicts that the fraction of time devoted to each letter of a word is kept constant and is independent of the total word duration [11,12]. When both these principles are fulfilled, relative and total execution time are approximately constant between different word sizes or speeds. Both these principles are reported to be altered in handwriting production among pupils with dysgraphia having dyslexia comorbidity [13]. Indeed, when asked to write a word on a piece of paper affixed on a digitizing tablet at different speeds and dimensions, primary school children affected by learning disabilities were not able to adapt their speed to task requirements, showing difference from their typically developed peers.

Experts stress the concept that pupils with dysgraphia tend to write every single letter faster than typically developing peers, with greater pauses and poorer accuracy [2]. Such observations suggest an alteration of the *speed-accuracy tradeoff* principle, which states that the more accurate the task, the longer it takes to accomplish it. Smits-Engelsman et al [14] reported that children with learning disabilities were less accurate in continuous cyclical fast aiming tasks, whereas they performed similarly to their peers when paced by an external “go” signal. The speed-accuracy tradeoff alteration in this group seems therefore related to continuous open loop activities, such as handwriting tasks. A model used to assess the speed-accuracy tradeoff during continuous movements is the *steering law* [15], a continuous formulation of the Fitts’ law [16] with a shift of the paradigm from pointing to path steering. Such a law was tested with different geometrical constraints, but was never applied to handwriting.

To meet the second requirement, uncovering the laws underlying handwriting in drawing is crucial. This would allow the detection of potential difficulties before the acquisition or at least before the complete maturation of the writing gesture. Previous studies on isochrony [17,18] confirmed that such a principle is characteristic of gesture production, thus allowing its study in drawings. As for homothety, an attempt of proving its presence before the complete maturation of handwriting was made by Pagliarini et al [19]. They investigated homothety during handwriting in children from the first to the fifth grade, revealing that the principle holds overall, but a deviation was observed for younger children. This finding leaves room for a further investigation on homothety at a preliteracy age.

Finally, to meet the third requirement, leveraging standard consumer technology is key to allowing easy access to the screening process, as reported in other fields [20]. In the scientific literature, the classical setup for quantitative and reliable handwriting evaluation involves digitizing tablets, which are still far from being deployed in nonclinical environments. Indeed, they are more expensive and less general purpose than standard consumer technologies, such as tablets. Moreover, data recording and fruition require specialized software, preventing the acquisition protocol from being straightforward to nonexpert users. For these reasons, tablet-based serious games have been recently proposed to both test perceptual motor skills in primary school children with and without suspected learning difficulties [21,22] and propose preliminarily treatments for dysgraphia [23]. Indeed, the use of tablet-based solutions entails many advantages like the opportunity of introducing encouraging feedback on the user’s performance and the possibility of accomplishing increased interaction and more dynamic scenarios. Such features contribute to achieving a greater engagement, which is key especially for children’s application. However, such a technology must be validated for the study of handwriting, since the tablet surface can affect writing performance. Difficulties in changing the writing support are particularly evident in children [24], and we must assure that a change in the writing support does not alter the handwriting laws that hold for the pen-and-paper standard approach.

### Objectives

Given the highlighted needs, we devised and developed *Play Draw Write*, a tablet app to investigate handwriting through serious games designed to test isochrony, homothety, and speed-accuracy tradeoff principles both in word writing and symbol drawing. We proposed the following three goals: (1) to investigate if these writing principles hold when writing on a tablet surface; (2) to verify that symbol drawing can anticipate the detection of potential difficulties at the preliteracy stage; and (3) to investigate how writing-related skills and principles change with gesture acquisition.

## Methods

### Materials

**Figure 1:**
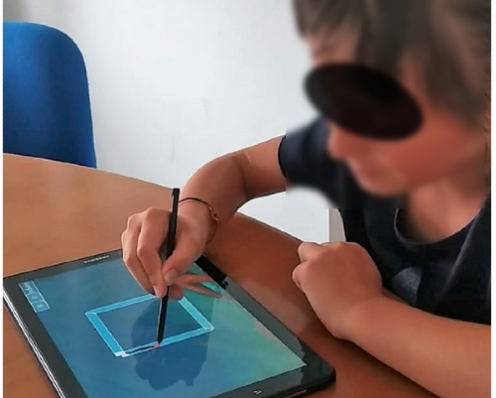
Experimental setup. A kindergartner is executing one of the exercises on the tablet with an S-Pen.

The hardware chosen for the experiment was Samsung Galaxy Tab A with an S-Pen (Figure 1). The S-Pen with a rubber tip was used to better mimic the paper-and-pencil condition.

The app was developed in Unity 2018.3.2f1 following the principles of theory-driven evidence-based serious games for health [25,26]. We set the sampling rate at 50 Hz, as the frequency content of the recorded movement was expected to be less than 22 Hz [27].

**Figure 2:**
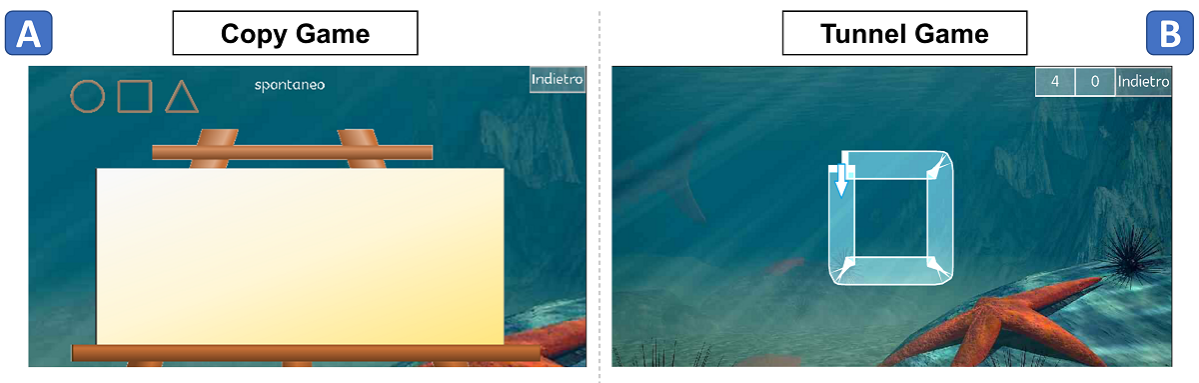
App interface. A: copy game; B: tunnel game.

The app *Play Draw Write* presented the following two exercises: *copy game* and *tunnel game*, which can be seen in Multimedia Appendix 1 and Multimedia Appendix 2, respectively. The copy game was aimed at testing both isochrony and homothety. We presented the participants an interface with an empty canvas and an example of the word and symbols they had to copy, together with the execution modality (Figure 2A). At the end of each execution, a new empty canvas appeared on the screen. As for the word, “mele” (“apples” in Italian) was chosen since it contains the letters E and L, which are useful to test praxical abilities [28]. As for the symbols, following the Denver test [29], a circle, square, and triangle were chosen. In addition, a sequence of these symbols was also used to test for homothety in symbols.

The tunnel game was aimed at testing the speed-accuracy tradeoff for both words and symbols. Our purpose was to detect the adaptability to different task requirements (ie, accuracy). According to the steering law, accuracy is defined by the index of difficulty (ID) as follows:

ID = A / W (**1**)

where W is the width between the borders of the path and A is the amplitude of the gesture (ie, perimeter for symbols and the path length for words, both measured in the center of the path). We combined A and W to obtain five different IDs for words and five for symbols, following clinicians’ guidelines (Multimedia Appendix 3). We presented the participants an interface with symbol- and word-shaped paths with different IDs (Figure 2B). An arrow indicated the direction to follow for symbol steering. If the border of the tunnel was crossed, a noise alerted participants to correct themselves. At the end of each execution, a new word/symbol appeared on the screen, with different accuracy constraints. Both for words and symbols, tutorials were provided to familiarize with the task. In the tutorial, a cartoon hand reproduced the exercise. Regarding words, paths were shaped as the word “ele.” Regarding symbols, paths were shaped as circles and squares. To prevent fatigue, the triangle was discarded from the tunnel game, as it is known to be the last symbol learned by children [29].

### Participants and Protocol

Data collection was performed on children attending the third grade of a primary school and the last year of a kindergarten in the province of Como, Italy. Target ages were selected as handwriting is mastered in third grade [7] and not yet learned in kindergarten. The inclusion criteria were as follows: both right and left handedness, and normal graphical abilities for kindergarten children, according to teachers’ judgement. The exclusion criteria were as follows: known cognitive or sensory-motor problems and learning disability diagnosed for primary school children. All children who attended the partner schools and met the inclusion criteria were invited to participate in the study.

A minimum sample size of 10 children per group was computed on the basis of previous experiments in the field of the speed-accuracy tradeoff [30]. We considered 80% power, a first-type error of 5%, and a drop-out rate of 20%.

**Figure 3:**
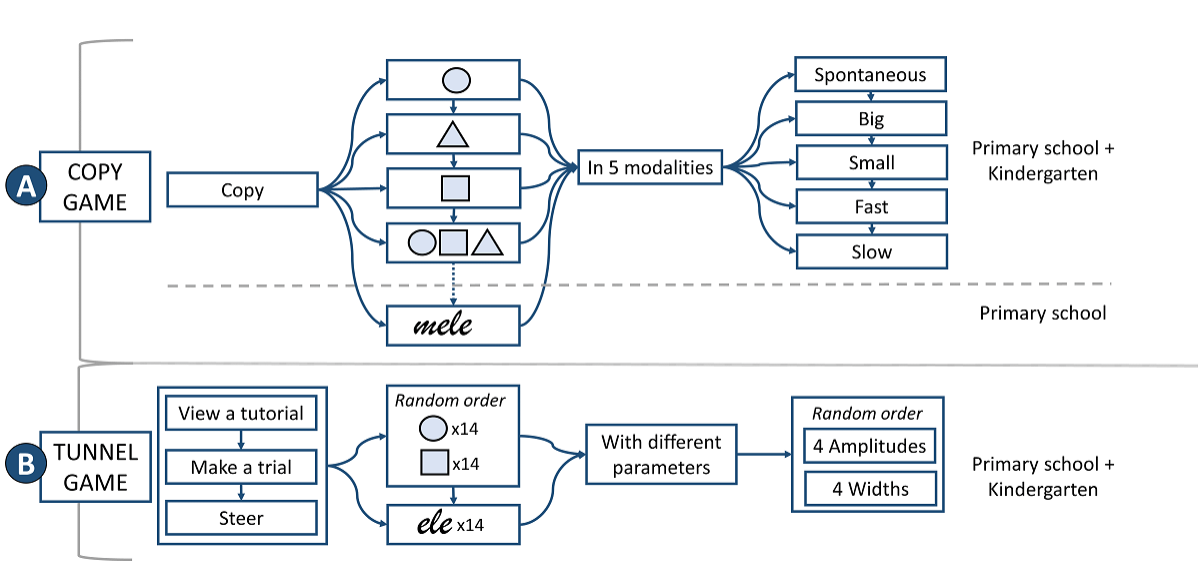
Study protocol. A: copy game; B: tunnel game.

We collected data at the end of the school year. Acquisition took place in a quiet room with the child comfortably seated. The complete protocol (Figure 3) duration was 15 to 30 minutes, according to the ability of the children.

In the copy game, the instructions were to copy the word/symbol shown in the upper part of the screen, in the indicated modality (Figure 2A), as in previous studies on similar tasks [13]. We also orally specified the modality, as kindergartners cannot read yet. Word copying was executed by primary school children only, as kindergartners cannot write yet. In this exercise, we specified that they could use their own handwriting style, as trying to copy in an unnatural handwriting would have biased the results. In the tunnel game, during the tutorial, children were instructed to steer the path twice for symbols and once for words, as fast as they could, without crossing the external border. If they accidentally drew outside the path, they were encouraged to correct themselves, without stopping the execution. The instruction gave equal importance to speed and accuracy, as per the theory of constant throughput in speed-accuracy tradeoff [31].

The experimental procedure was approved by the Ethics Committee of Politecnico di Milano (n.10/2019). Written consent was received from school deans, parents, and primary school children.

### Data Analysis and Statistics

Data analysis was performed in Matlab R2018b (MathWorks) and R 3.3.3 (R Development Core Team). Significance was set at .05. Given the small sample size, nonparametric statistics were selected for all tests.

#### Copy Game

First, instruction compliance was tested using a Wilcoxon matched paired test comparing the trajectory length between the *big* and *small* modalities.

To test isochrony, separately for words and symbols, we considered between-condition differences in the average execution speed. To be compliant with the isochrony principle, we expected the *big* and *fast* modalities to be executed faster than the *small* and *slow* modalities. We first verified the validity of the law in primary school children (words and symbols) and then extended it to kindergartners to test the potential influence of gesture acquisition.

We considered speed, rather than time, to normalize the execution time for the size, as it was self-selected. For both words and symbols, we discarded the first and last 5% of the trajectory to avoid border effects. Thereafter, we computed speed as the ratio between the discrete difference between adjacent coordinates and the corresponding timestamps. We filtered the result with a 10-Hz low-pass filter, and we averaged the speed over the entire path to obtain a single value per execution. To test the effect of the modality on speed, we used Friedman and Bonferroni *post-hoc* tests.

To understand if the primary school and kindergarten groups approached the exercise differently, we performed, separately for each symbol, a Mann-Whitney *U* test with the *spontaneous* modality speed as the dependent variable and the age group as the independent variable.

To test for homothety on both word and symbol sequences, we computed the percentage of time (*fraction time*) dedicated to each element of the sequence. For cursive words, the lowest point between two letters was considered as the transition point. Concerning symbols, the first and last frames the pen was on the screen were considered as the beginning and the end of a symbol, respectively. A Friedman test was performed separately on words and symbols (independent variable: modality; dependent variable: fraction time). In case of significance, Bonferroni *post hoc* was executed. No effect of the modality was expected.

#### Tunnel Game

To study the speed-accuracy tradeoff during handwriting, the steering law was adopted. The steering law predicts a linear relationship between movement time (MT) and ID as follows:

MT = a + b ID (**2**)

**Figure 4:**
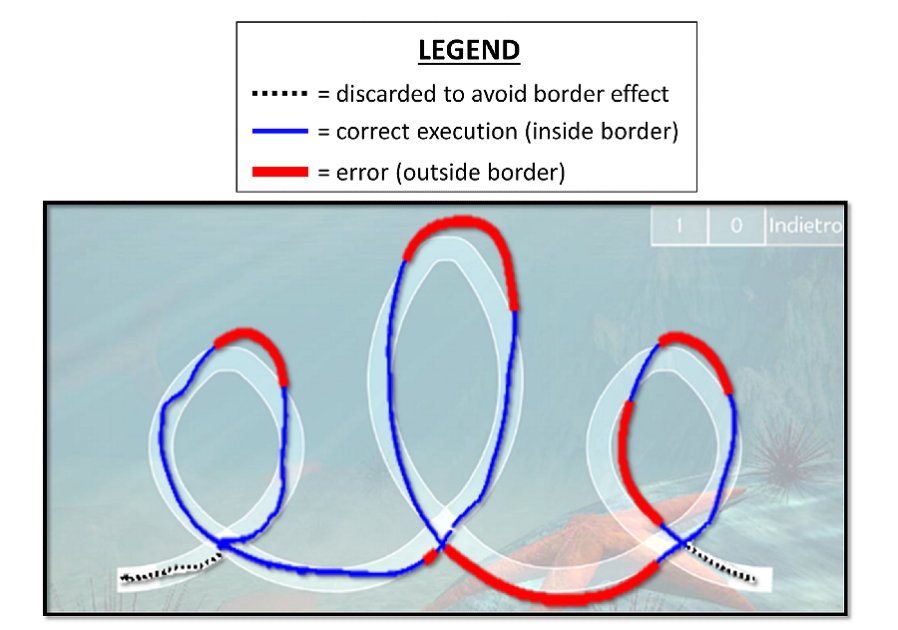
Speed-accuracy tradeoff exercise. The transparent background represents the app interface.

To compute MT in the word “ele,” we considered the timestamp difference between the first “*E*” crossing and the last “*E*” crossing (Figure 4).

To compute MT in symbols, we considered the central part of the execution as follows: for the circle, we discarded the first and last semicircles, leaving a complete central circle; for the square, we discarded the first two and the last two sides, leaving a complete central square. We discarded trials where more than 40% of the path was outside the borders [31], in respect to the total length of the trace (Figure 4; error rate: 42.76%).

Separately for the word and the two symbols, we calculated the *global MT*, that is, the median movement time across all IDs, for each subject. For the word and the two symbols, we computed a linear regression between IDs, as an independent variable, and MT, as a dependent variable, for each subject separately. For each regression, we computed the R^2^ and we checked for regression significance that implies the validity of the law. Moreover, we evaluated significant fittings using the root mean square error (RMSE) of regression as an index of the goodness of fit. Significant fittings only were used to compute the inverse of the regression line slope (the index of performance [IP]), which was computed as follows:

IP = 1 / b (**3**)

The IP is an index of adaptation to a task’s accuracy requirements [16]. We tested for differences in MT, RMSE, and IP, considering both age group (Mann-Whitney test) and symbols (Friedman test) as independent variables. When significance was reached, a Bonferroni *post-hoc* test was performed.

## Results

### Participants

We enrolled 15 right-handed primary school children (7 males and 8 females; mean age 8.8 years, SD 0.3) and 19 kindergartners (9 males and 10 females; 2 left handed; mean age 5.9 years, SD 0.4).

### Data Analysis and Statistics

**Figure 5:**
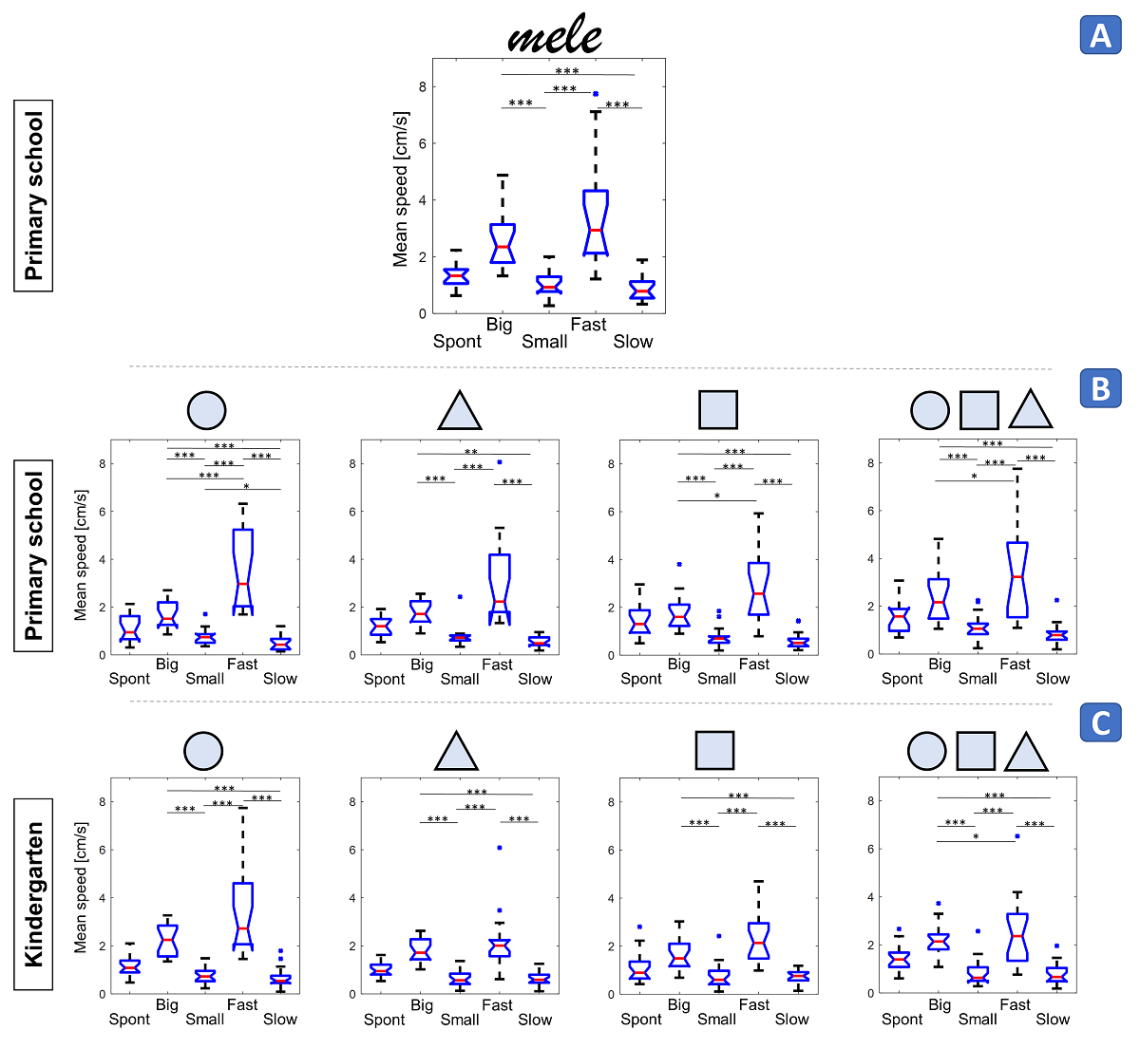
Isochrony. A: word, primary school; B: symbols, primary school; C: symbols, kindergarten. X-axis: execution modality; Y-axis: mean speed as box-and-whiskers plots. The red horizontal line is the median, the notch is its 95% CI, and the box is the IQR. Asterisks for between-condition differences involving the spontaneous modality are not reported for clarity purposes.*P<.05, **P<.005, ***P<.001.

**Figure 6:**
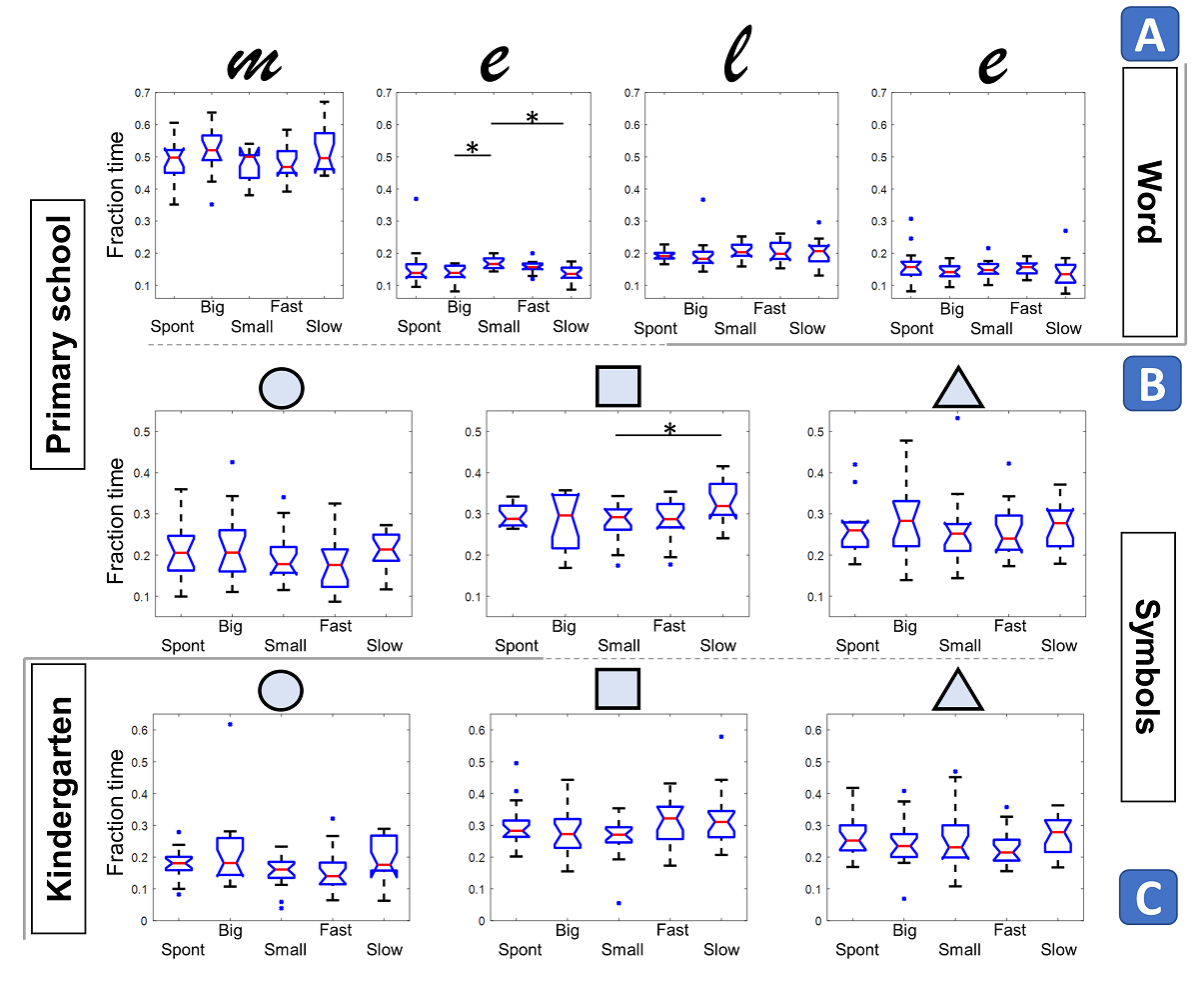
Homothety. A and B: primary school; C: kindergarten. A: word; B and C: symbols. Each subplot represents one element of the sequence of letters or symbols. X-axis: execution modality; Y-axis: fraction time, in normalized unit (1 represents the total execution time) as box-and-whiskers plots. The red horizontal line is the median, the notch is its 95% CI, and the boxes represent the IQR. *P<.05, **P<.005, ***P<.001.

**Figure 7:**
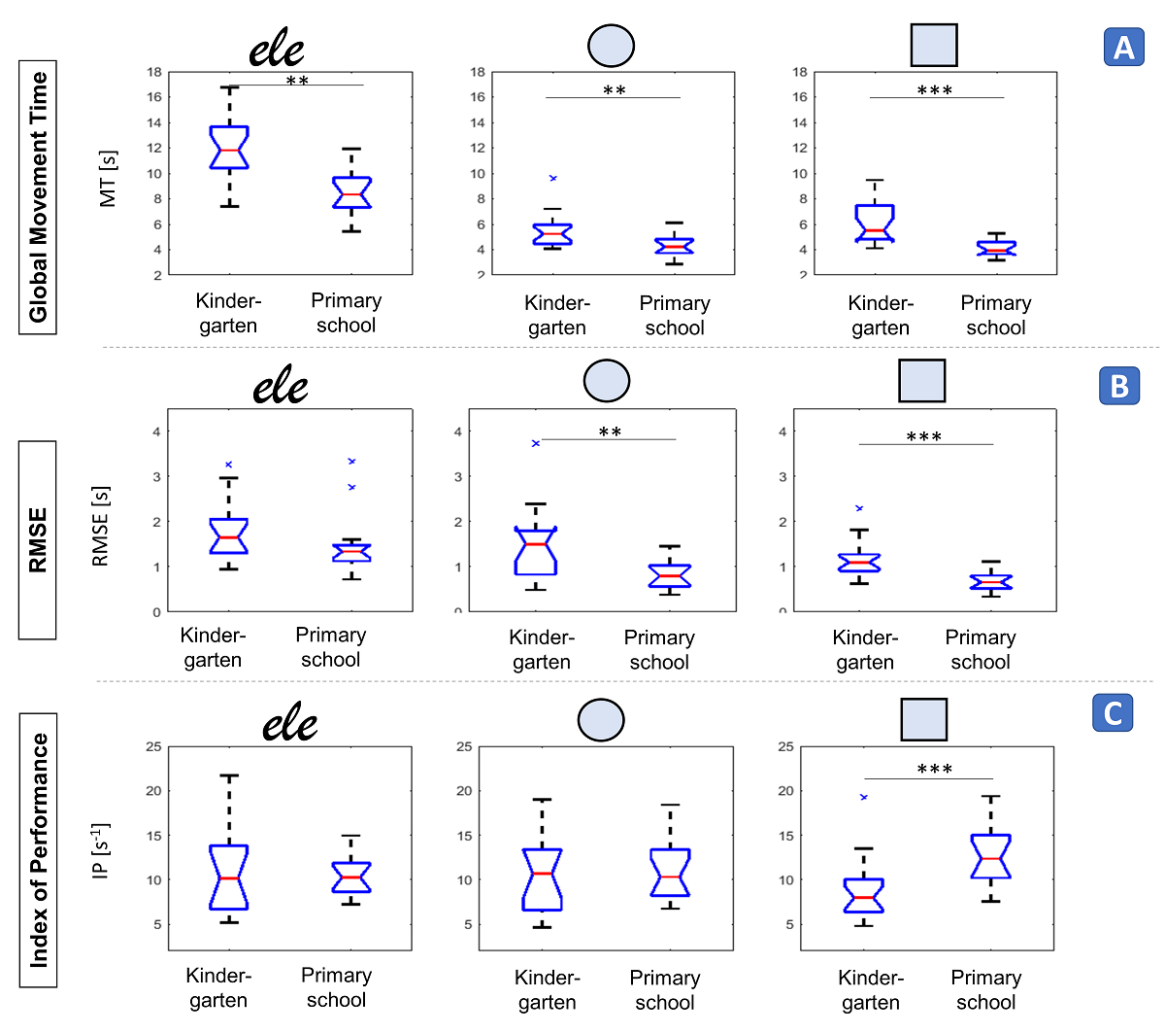
Speed-accuracy tradeoff. A: global movement time (ie, the median movement time computed across all indexes of difficulty). B: root mean square error (RMSE) (ie, the goodness of fit of the steering law). C: index of performance (ie, the inverse of the regression line slope). Data are divided by symbol/word and age group. *P<.05, **P<.005, ***P<.001.

Test results are reported in Multimedia Appendix 4 and Multimedia Appendix 5, and in Figures 5-7.

#### Copy Game

Concerning the copy game, instruction compliance was confirmed by all subjects in all the tests. Indeed, the trajectory in the *big* modality was always significantly longer than that traced in the *small* modality (*P*<.001, Multimedia Appendix 4, *exercise compliance*).

The speed execution of the word “mele” (Figure 5A; Multimedia Appendix 4, *isochrony*) reported a significant effect of the modality (*P*<.001) in primary school children. In particular, speed in the *big* modality was greater than in the *small* (*P*<.001) and *slow* modalities (*P*<.001), and that in the *fast* modality was greater than in the *small* (*P*<.001) and *slow* (*P*<.001) modalities.

A significant effect of the modality was also found for symbol speed execution in primary school children (*P*<.001) (Figure 5B). The speed in the *big* modality was greater than in the *small* (*P*<.001 in all cases) and *slow* (*P*=.002 for triangles, *P*<.001 in all other cases) modalities, and that in the *fast* modality was greater than in the *small* (*P*<.001 in all cases) and *slow* (*P*<.001 in all cases) modalities.

The copy game (Figure 5C) showed a significant effect of the modality on speed for all symbols (*P*<.001) and for kindergartners. In particular, speed in the *big* modality was greater than in the *small* (*P*<.001 in all cases) and *slow* (*P*<.001 in all cases) modalities, and that in the *fast* modality was greater than in the *small* (*P*<.001 in all cases) and *slow* (*P*<.001 in all cases) modalities.

When investigating possible mean speed differences in the *spontaneous* modality between the two age groups, the Mann-Whitney *U* test did not reveal a significant effect of age for any of the symbols (circles: *P*=.55; triangles: *P*=.10; squares: *P*=.44; sequences: *P*=.50; Multimedia Appendix 4, *developmental trend*).

When analyzing the fraction time of letters in primary school children (Figure 6A; Multimedia Appendix 4, *homothety*), no significant between-condition differences were highlighted for *M* (*P*=.16), *L* (*P*=.20), and the last *E* (*P*=.21), thus supporting the principle of homothety. A significant effect of the modality emerged only for the first *E* letter (*P*=.004). Post-hoc tests revealed that the fraction time for the first *E* letter in the *small* modality was significantly higher than in the *big* (*P*=.006) and *slow* (*P*=.009) modalities.

As for the fraction time in the sequence of symbols (Figure 6B), one primary school child was removed from the *big* modality dataset owing to erroneous execution. No fraction time differences emerged, with the only exception of squares between the *small* and *slow* modalities (*P*=.03).

As for the sequence of symbols in the kindergartners (Figure 6C), the modality had no effect on fraction time for squares (*P*=.06) and triangles (*P*=.30). We found a significant effect of the modality for circles (*P*=.02), which was not supported by the post-hoc assessment.

#### Tunnel Game

Among all trials, two kindergartners were not able to comply with the instructions during word steering, and thus, their data were removed.

Global MT (Figure 7A; Multimedia Appendix 5, *developmental trend*) was always significantly higher in kindergartners than in primary school children (word: *P*=.001; circles: *P*=.003; squares: *P*<.001).

In the primary school group, the linear fitting between ID and MT was significant for all children, except one in the square task. In the kindergarten group, all children obtained significant fitting for the square, whereas 15/19 circles and 15/17 word fittings were significant (*P*<.05, R^2^>0.28, 12 degrees of freedom; Multimedia Appendix 5, *exercise compliance*).

The RMSE of symbol fitting (Figure 7B; Multimedia Appendix 5, *developmental trend*) was significantly lower (better fitting) for primary school children than for kindergartners (circle: *P*=.02; square: *P*<.001). The between-age difference in the RMSE of word fitting did not reach significance (*P*=.06), but the same trend was visible. RMSE differences between the word and symbols emerged in the primary school group (*P*<.001; Multimedia Appendix 5, *motor strategy differences*), but not in the kindergarten group (*P*=.08). In the primary school group, words fitted significantly worse than circles (*P*=.001) and squares (*P*<.001), but we did not find any significant difference between circles and squares (*P*=.65).

The IP (Figure 7C) showed a significant effect of age for squares (*P*<.001), but not circles (*P*=.65) or words (*P*=.80). No differences between the word and symbols emerged (primary school: *P*=.08; kindergarten: *P*=.13).

## Discussion

A tablet-based app to investigate handwriting difficulties through serious games was developed and validated. Standard consumer technology, which does not require supervision and is suitable for home or school use, was leveraged to increase the usability and accessibility of the devised tool. The app was designed to quantitatively characterize the three principles underlying handwriting of isochrony, homothety, and speed-accuracy tradeoff in not only word writing but also symbol drawing, with the final aim of anticipating the screening procedure for handwriting alterations in the preliteracy stage.

To verify that isochrony, homothety, and speed-accuracy tradeoff hold on a tablet surface during word writing, the app was tested on 15 typically developing third graders. Results showed that the tablet surface did not affect the presence of these laws for cursive word writing.

Specifically, isochrony was verified, as writing bigger always increases speed and writing smaller has the opposite effect. This confirms the results reported by Pagliarini et al [13], where 39 typically developing primary school children showed isochrony when writing on a piece of paper affixed on a professional digitizer. Our results extend the application domain of their findings, showing that a change in the writing support from a professional digitizer to a commercial tablet does not affect the presence of the isochrony law.

Additionally, the homothety principle holds on the tablet surface, as it was respected in 38 out of 40 cases during cursive writing. Neither writing size nor speed showed a relevant effect on the percentage of time dedicated to each letter with respect to the total word duration, with the only exception of one letter in the *small* modality, which significantly differed from both the *big* and *slow* modalities. This small exception may be ascribed to the nature of the cursive script. As letters are smoothly joined, different segmentation techniques might bring different outcomes. Indeed, our results on the tablet are in line with and further enhance previous work [13], which reported a higher number of exceptions of the homothety principle for the cursive script on a digitizer.

Finally, the speed-accuracy tradeoff principle was confirmed while writing on the tablet surface, since the steering law on words reached significance for 14 out of 15 primary school children. Importantly, these results support our proposal to leverage the steering law to study the speed-accuracy tradeoff during handwriting, which was never performed previously.

The above results support the use of tablet technology to quantitatively assess handwriting production. This is an important achievement, as previous research revealed that changing the writing surface is challenging for writers. Although literature reports an increase in writing speed resulting from the low friction coefficient of the tablet surface [24], this work proves that possible differences in specific parameters are not reflected in the violation of more high-level handwriting laws.

To detect possible handwriting alterations at an early stage, we investigated whether the principles underlying word writing hold also for symbol drawing in 15 typically developing third graders. Concerning isochrony, we found that it held for symbols. Indeed, isochrony-related speed modulation was observed for all symbols.

Results on homothety further support the possibility to replace a word with a sequence of symbols. Indeed, homothety regarding symbols was confirmed in 29 out of 30 cases. Our results showed that the principle of homothety is even stronger for a sequence of symbols than for a cursive word. An analogy can be found with the work of Pagliarini et al [13], where homothety was verified for the capital script, but showed some exceptions for the cursive script. We argue that a sequence of capital script letters and a sequence of symbols are similar from both a planning and an execution point of view, thus making the two exercises interchangeable.

As for the speed-accuracy tradeoff for symbols, the linear regression of MT on ID was found to be relevant in 29 out of 30 trials.

Thus, we can conclude that symbols are valid candidates to substitute words and anticipate the study of possible violations of the principles, typical of dysgraphia.

Finally, we tested our app among 19 kindergartners to investigate if and how handwriting maturation affects the skills and laws underlying handwriting. Speed modulation and fraction time preservation were confirmed among kindergarteners during symbol drawing in 100% of cases, supporting that adherence to the isochrony and homothety principles when writing on a tablet surface does not develop as a consequence of mastering handwriting [17-19]. Previous work [19] reported some exceptions to the homothety principle during block letter writing, especially in younger children. In light of our results, we claim that a sequence of symbols is more suitable than a block letter word when investigating homothety at a preliteracy age. Indeed, symbol drawing has the advantages of being acquired years before handwriting and being language independent [32].

Among kindergartners, the speed-accuracy tradeoff was studied while steering both symbols and a cursive word, since we hypothesized that path guidance was a valid help in task execution even before writing acquisition. Our findings showed that the linear regression of MT on ID was significant in the majority of cases (*P*<.05, R^2^>0.28, 12 degrees of freedom, 15/17 for words and 34/38 for symbols). Our results support that this principle is innate both in path steering and in handwriting, and can be potentially used as a screening tool at a preliteracy age.

The steering law parameters revealed a developing trend with age. In particular, kindergartners needed more time to complete the speed-accuracy exercises than older children. This result seems to contradict the group invariance of the average speed obtained when copying symbols (copy game in the *spontaneous* modality). We can conjecture that sticking to complex tasks is more demanding for kindergarteners, but they do not have any specific difficulty in free execution. This consideration is further supported when analyzing the IP during square tracing. Kindergartners achieved significantly lower results than primary school children (*P*<.001), showing that they need a bigger adaptation effort (ie, longer MT) to react to different difficulty constraints. The square symbol seems to be the most adequate to disclose such between-group difference, probably since it is the most similar to the steering law original formulation [15]. The same age-related trend was found in previous work on the speed-accuracy tradeoff in the discrete domain [30,33], supporting the use of the speed-accuracy tradeoff principle as an index of maturity of motion control. The same law was also proven to successfully discriminate between healthy and pathological conditions (eg, dystonia [34], and Huntington and Parkinson diseases [35]). In this work, we further proved that steering’s IP is useful to differentiate between ages, and we speculate that it has the potential to differentiate between pathological and normal handwriting as well. Therefore, we plan to continue our experiments on a group of pupils with dysgraphia to further prove the opportunity to exploit our tool to support the identification of handwriting abnormalities.

Finally, a higher RMSE emerged for kindergartners during symbol drawing, suggesting that adherence to the steering law is a process that is refined with age. In primary school children only, the RMSE for word tracing was significantly higher than that for symbol tracing (*P*=.001 for word-circle comparison, *P*<.001 for word-square comparison). This result can be explained by the different kinds of movements required to produce a letter. Indeed, mastered handwriting can be considered open loop [36], and this aspect might alter the kinematic pattern modelled by the steering law. To support such a hypothesis, the lack of differences in kindergartners suggests that they approached words and symbols similarly, as they did not learn or automate handwriting yet. Further experiments can clarify this aspect.

In conclusion, we proposed a simple tool to quantify the degree of deviation from physiological handwriting, which could help with the objective detection of dysgraphic alterations. The tool exploits serious games to assess important rhythmic principles of handwriting, such as isochrony, homothety, and speed-accuracy tradeoff. It does not require any professional equipment or professional supervision, making the assessment simple and accessible in nonclinical environments. Importantly, we proved that it is possible to start evaluating characteristics underlying handwriting in preschoolers through the use of symbols, which makes the evaluation language independent. Eventually, the tool can provide interesting insights on graphomotor abilities correlated with handwriting and can be used to monitor their evolution over time. Thus, the proposed tool paves the way toward a new model of care. It includes the screening of preliteracy skills to identify potential difficulties before they arise and offers continuous, accessible, and low-cost monitoring of their evolution.

## Appendix

Multimedia Appendix 1 Copy game: sequence.

Multimedia Appendix 2 Tunnel game: word.

Multimedia Appendix 3 Index of difficulty calculation.

Multimedia Appendix 4 Statistical test results for the copy game.

Multimedia Appendix 5 Statistical test results for the tunnel game.

## Abbreviations

CI: confidence interval
ID: index of difficulty
IP: index of performance
IQR: interquartile range
MT: movement time
RMSE: root mean square error
