# Supplementary material for: A Tablet App for Handwriting Skill Screening at the Preliteracy Stage: Instrument Validation Study
Source: JMIR Serious Games. 2020 Oct 22;8(4):e20126. doi: 10.2196/20126 (PMC7644384; doi:10.2196/20126)
Supplement: Multimedia Appendix 3 [file games_v8i4e20126_app3.pdf]

Indexes of Difficulty calculation
Amplitudes (path lengths) are presented in columns, and widths in rows.
In the first part, values refer to words, in the second part, values refer to symbols.

**Word ID**		**Amplitude, cm**			
		**26.8**	**37.8**	**52.2**	**74.9**
**Width, cm**	**0.4**	66.7	93.9	131.2	-
	**0.6**	47.2	66.7	93.9	131.2
	**0.8**	33.8	47.2	66.7	93.9
	**1.1**	-	33.8	47.2	66.7

**Symbol ID**		**Amplitude, cm**			
		**14.8**	**20.7**	**29**	**40.6**
**Width, cm**	**0.5**	29.6	41.4	58.0	-
	**0.7**	21.1	29.6	41.4	58.0
	**1**	15.1	21.1	29.6	41.4
	**1.4**	-	15.1	21.1	29.6

This is a Multimedia Appendix to a full manuscript
published in JMIR Serious Games, titled
“A Tablet App for Handwriting Skill Screening at the Preliteracy Stage:
Instrument Validation Study”
