# Supplementary material for: A Tablet App for Handwriting Skill Screening at the Preliteracy Stage: Instrument Validation Study
Source: JMIR Serious Games. 2020 Oct 22;8(4):e20126. doi: 10.2196/20126 (PMC7644384; doi:10.2196/20126)
Supplement: Multimedia Appendix 4 [file games_v8i4e20126_app4.pdf]

COPY GAME

EXERCISE COMPLIANCE

Wilcoxon matched paired test: modality effect on trajectory length, *Big* VS *Small*

	Primary school			Kinsergarten		
	Big, cm	Small, cm	p-value	Big, cm	Small, cm	p-value
	median (IQR)			median (IQR)		
Word	65.6 (11.2)	14.9 (6.1)	*P* <.001	-	-	
Circle	29.7 (9.5)	6.3 (3.6)	*P* <.001	27.2 (14.5)	4.2 (3.5)	*P* <.001
Triangle	32.4 (13.9)	8.0 (3.0)	*P* <.001	25.4 (11.6)	5.2 (4.7)	*P* <.001
Square	37.8 (8.7)	7.3 (3.1)	*P* <.001	31.4 (10.4)	7.4 (6.3)	*P* <.001
Sequence	71.8 (17.0)	20.7 (8.6)	*P* <.001	73.1 (25.3)	18.3 (13.3)	*P* <.001

ISOCHRONY

Friedman and Bonferroni *post hoc* : modality effect on average speed

Word *P* <.001

Primary school

Post hoc	*Spont.*	*Big*	*Small*	*Fast*
Big	*P* <.001	-		
Small	*P* =.39	*P* <.001	-	
Fast	*P* <.001	*P*= .51	*P* <.001	-
Slow	*P* =.11	*P* <.001	*P* >.99	*P* <.001

Circle *P* <.001

	Primary School				Kindergarten			
Post hoc	Spont.	Big	Small	Fast	Spont.	Big	Small	Fast
Big	*P* <.001	-			*P* <.001	-		
Small	*P* =.12	*P* <.001	-		*P* =.01	*P* <.001	-	
Fast	*P* <.001	*P* <.001	*P* <.001	-	*P* <.001	*P* =.10	*P* <.001	-
Slow	*P* <.001	*P* <.001	*P* =.006	*P* <.001	*P* =.004	*P* <.001	*P* >.99	*P* <.001

Triangle *P* <.001

	Primary School				Kindergarten			
Post hoc	*Spont.*	*Big*	*Small*	*Fast*	*Spont.*	*Big*	*Small*	*Fast*
Big	*P* =.006	-			*P*= .001	-		
Small	*P* =.39	*P* <.001	-		*P* <.001	*P* <.001	-	
Fast	*P* <.001	*P* =.13	*P* <.001	-	*P*= .001	*P* >.99	*P* <.001	-
Slow	*P* =.08	*P*= .002	*P* =.51	*P* <.001	*P* <.001	*P* <.001	*P* >.99	*P* <.001

Square *P* <.001

	Primary School				Kindergarten			
Post hoc	Spont.	Big	Small	Fast	Spont.	Big	Small	Fast
Big	*P* =.006	-			*P* <.001	-		
Small	*P* =.08	*P* <.001	-		*P* =.01	*P* <.001	-	
Fast	*P* <.001	*P* =.02	*P* <.001	-	*P* <.001	*P* =.87	*P* <.001	-
Slow	*P* =.004	*P* <.001	*P* =.29	*P* <.001	*P* <.001	*P* <.001	*P* >.99	*P* <.001

Sequence *P* <.001

	Primary School				Kindergarten			
Post hoc	Spont.	Big	Small	Fast	Spont.	Big	Small	Fast
Big	*P* =.16	-			*P* <.001	-		
Small	*P* =.008	*P* <.001	-		*P*= .006	*P* <.001	-	
Fast	*P* <.001	*P* =.03	*P* <.001	-	*P* <.001	*P*= .005	*P* <.001	-
Slow	*P* <.001	*P* <.001	*P* =.74	*P* <.001	*P*= .002	*P* <.001	*P* >.99	*P* <.001

DEVELOPMENTAL TREND

Mann-Whitney U test: age effect on *Spontaneous* speed

	Circle	Triangle	Square	Sequence
P -value	*P* =.55	*P* =.10	*P* =.44	*P* =.50

HOMOTHETY

Friedman and Bonferroni *post hoc* : modality effect on fraction time

Word

	M	E1	L	E2
P -value	*P* =.16	*P* =.004	*P* =.20	*P* =.21

E1 post hoc

	Spont.	Big	Small	Fast
Big	*P* >.99	-		
Small	*P* >.99	*P* =.006	-	
Fast	*P* >.99	*P* =.18	*P* =.83	-
Slow	*P* >.99	*P* >.99	*P* =.009	*P* =.15

Sequence, primary school

	Circle	Square	Triangle
P -value	*P* =.52	*P* =.04	*P* =.30

Square post hoc

	Spont.	Big	Small	Fast
Big	*P* >.99	-		
Small	*P* >.99	*P* >.99	-	
Fast	*P* >.99	*P* >.99	*P* >.99	-
Slow	*P* =.18	*P* =.26	*P* =.03	*P* =.26

Sequence, kindergarten

	Circle	Square	Triangle
P -value	*P* =.02	*P* =.06	*P* =.30

Circle post hoc

	Spont.	Big	Small	Fast
Big	*P* >.99	-		
Small	*P* =.29	*P* =.07	-	
Fast	*P* =.55	*P* =.08	*P* >.99	-
Slow	*P* >.99	*P* >.99	*P* =.23	*P* =.87

This is a Multimedia Appendix to a full manuscript
published in JMIR Serious Games, titled
“A Tablet App for Handwriting Skill Screening at the Preliteracy Stage:
Instrument Validation Study”
