# Supplementary material for: A Tablet App for Handwriting Skill Screening at the Preliteracy Stage: Instrument Validation Study
Source: JMIR Serious Games. 2020 Oct 22;8(4):e20126. doi: 10.2196/20126 (PMC7644384; doi:10.2196/20126)
Supplement: Multimedia Appendix 5 [file games_v8i4e20126_app5.pdf]

TUNNEL GAME

EXERCISE COMPLIANCE

Steering law: linear regression significance (R2 and number of valid trials)

Primary school

	Word		Circle		Square	
Subject	R2	n	R2	n	R2	n
1	0.79	14	0.94	14	0.64	14
2	0.85	14	0.34	14	0.91	14
3	0.83	14	0.48	14	0.61	14
4	0.78	14	0.33	14	0.84	14
5	0.61	14	0.84	14	0.79	14
6	0.94	14	0.92	14	0.74	14
7	0.87	14	0.69	14	0.84	14
8	0.74	14	0.68	13	0.37	14
9	0.91	14	0.59	14	0.56	14
10	0.87	14	0.67	14	0.63	14
11	0.89	14	0.87	14	0.86	14
12	0.93	14	0.90	14	0.83	14
13	0.47	14	0.59	14	0.14	14
14	0.96	14	0.88	13	0.90	14
15	0.87	14	0.83	14	0.75	14

Kindergarten

	Word		Circle		Square	
Subject	R2	n	R2	n	R2	n
1	0.71	7	0.58	14	0.89	14
2	0.81	14	0.32	14	0.77	14
3	-	0	0.84	14	0.77	14
4	0.2	14	0.40	12	0.48	14
5	0.52	14	0.69	14	0.57	13
6	0.58	14	0.69	14	0.31	14
7	0.84	14	0.76	13	0.71	14
8	-	2	0.55	14	0.68	14
9	0.84	14	0.39	14	0.73	14
10	0.90	14	0.27	14	0.81	14
11	0.63	14	0.20	13	0.78	14
12	0.89	14	0.70	14	0.83	14
13	0.84	14	0.80	14	0.67	14
14	0.72	14	0.34	14	0.70	14
15	0.68	14	0.40	14	0.49	14
16	0.82	14	0.10	14	0.88	14
17	0.85	14	0.27	14	0.73	14
18	0.28	14	0.39	14	0.38	13
19	0.93	11	0.40	14	0.74	14

DEVELOPMENTAL TREND

Mann-Whitney U test: age effect on parameters

	Word	Circle	Square
MT	*P* =.001	*P* =.003	*P* <.001
RMSE	*P* =.06	*P* =.02	*P* <.001
IP	*P* =.80	*P* =.65	*P* <.001

MOTOR STRATEGY DIFFERENCES

Friedman test and Bonferroni post hoc: word or symbol effect on parameters

	Primary school				Kindergarten			
	Friedman	Post hoc			Friedman	Post hoc		
	Symbol effect	Word-Circle	Word-Square	Circle-Square	Symbol effect	Word-Circle	Word-Square	Circle-Square
MT	*P* =.001	*P* =.003	*P* =.01	*P* =.99	*P* =.003	*P* =.59	*P* <.001	*P* =.09
RMSE	*P* <.001	*P* =.001	*P* <.001	*P* =.65	*P* =.08	-	-	-
IP	*P* =.08	-	-	-	*P* =.13	-	-	-

This is a Multimedia Appendix to a full manuscript
published in JMIR Serious Games, titled
“A Tablet App for Handwriting Skill Screening at the Preliteracy Stage:
Instrument Validation Study”
